# Interactive cueing with walk-Mate for Hemiparetic Stroke Rehabilitation

**DOI:** 10.1186/1743-0003-9-58

**Published:** 2012-08-21

**Authors:** Takeshi Muto, Barbara Herzberger, Joachim Hermsdoerfer, Yoshihiro Miyake, Ernst Poeppel

**Affiliations:** 1 GRP - Generation Research Program, Humanwissenschaftliches Zentrum, Ludwig-Maximilians-Universitat München, Prof.-Max-Lange-Platz 11, 83646, Bad Tölz, Germany; 2 Tokyo Institute of Technology, G3-821, 4259, Nagatsuta, Midori-ku, Yokohama, Kanagawa 226-8502, Japan; 3 Aoyama Gakuin University, 5-10-1 O-505b, Fuchinobe, Chuo-ku, Sagamihara, Kanagawa, 252-5258, Japan; 4 EKN - Entwicklungsgruppe Klinische Neuropsychologie, Abteilung fuer Neuropsychologie, Krankenhaus München-Bogenhausen, Dachauer Strasse 164, D-80992, Munich, Germany; 5 Technische Universität München, Connolly strasse. 32, 80809, Munich, Germany; 6 Institute for Medical Psychology, Ludwig -Maximilians-Universitat München, Goethestrasse 31, D-80336, Munich, Germany

## Abstract

**Background:**

Many techniques that compensate for locomotion problems in daily life using externally controlled stimulation have recently been reported. These techniques are beneficial for effortlessly supporting patients’ locomotive functions, but the users of such devices must necessarily remain dependent on them. It is possible that some individuals with gait impairment may be prevented recovering locomotive function. From a rehabilitation viewpoint, it may therefore be supposed that ideally, devices that can be used in daily life to improve the locomotive functions of the body itself should be proposed.

**Methods:**

We evaluate the effectiveness of Walk-Mate, which has been used mainly as a gait compensation device, as a gait rehabilitation training device by analyzing improvement in locomotion before, during and after rehabilitation in hemiparetic patients and comparing it with a previous gait training method. Walk-Mate generates a model walking rhythm in response to a user’s locomotion in real time, and by indicating this rhythm using auditory stimuli, provides a technology that supports walking by reducing asymmetries and fluctuations in foot contact rhythm. If patients can use the system to learn a regulated walking rhythm, then it may also be expected to fulfil the functions of a gait rehabilitation training device for daily life.

**Results:**

With regard to asymmetry, significantly improvements were seen for compensatory movement during training using Walk-Mate, but improvements were not retained as rehabilitative results. Regarding fluctuations in the foot contact period, significant improvement was observed for compensatory movement during training and these significant improvements were retained as rehabilitative results. In addition, it became clear that such improvement could not be adequately obtained by the previously proposed training technique utilizing constant rhythmic auditory stimulation.

**Conclusions:**

Walk-Mate effectively compensated for locomotion problems of hemiparetic patients by improving gait rhythm both during and after training, suggesting that locomotive function can be effectively recovered in some patients. The interactive mechanism of Walk-Mate may be capable of simultaneously achieving the aims of gait compensation and gait rehabilitation training methods previously developed under individual frameworks. Walk-Mate is a promising technology for assisting the reintegration of disabled persons into society.

## Background

Many patients with cranial nerve damage suffer from motor paralysis and motor impairment. In particular, gait impairment that accompanies motor impairment of the lower extremities degrades locomotive function and presents a serious barrier to patients’ reintegration into society. This paper presents a new gait support technology aimed at patients with motor impairment of the lower extremities, and includes an evaluation of its effectiveness.

To date, two main types of treatment technologies have been presented for patients with gait impairment. One type involves techniques for complementing the gait function lost through impairment. These are generally known as gait compensation techniques, and are aimed at replacing the user’s impaired gait function by compensating their locomotive functions during daily life. The majority of such devices take the form of walking sticks and wheelchairs supporting patients with the aid of their own upper body motion. However, the development of other devices that provide compensation via external dynamic support has recently been advancing. Artificial legs with control systems [1,2], load-controlled gait assistance devices [3], power-assistive gait support machines [4-6], and powered suits [7,8] are some specific examples. These are all compensation technologies that support the elements of lower body motion (range of joint motion and physical strength) using actuators. Gait support using these devices assists humans by providing the control system functions of the mechanism itself unilaterally. However, since all the supportive technologies taking this form complement patients’ locomotive capabilities, the body itself must depend on the capabilities provided by the device. The users’ ability to generate a gait for themselves ceases to be essential, so it becomes difficult for patients to recover their own locomotive capabilities if they use such devices. While they may be considered effective at preventing disuse syndrome [9], and alleviating certain mechanical disorders, the recovery of the neurophysiological mechanisms needed to generate gait motion when using such devices remains difficult.

The other type of treatment technology includes devices aimed at restoring the user's locomotive capability by supporting gait rehabilitation training. In general, since this kind of training is planned and carried out by therapists using physical treatment methods, the objective of this kind of device is to support training according to the physical treatment method used. Training methods for improving the stability of locomotion [10,11] have been proposed involving training devices such as treadmills and stepping exercises, indicating rhythm aurally, or by visually indicating stimuli at uniform distances. In particular, it has been reported that as a result of such techniques, when patients with Parkinson's disease walked on a floor with a striped pattern, the periodic variation was significantly reduced [12]. Furthermore, Parkinson’s patients and stroke patients listening to a musical stimulus with a fixed tempo and rhythm demonstrated a significant improvement in the recovery of walking pattern [10,11]. Effectiveness of each of these techniques has been reported to realize safety regulated step timing and rhythm according to the user’s adaptation to an externally controlled environment. These methods are able to lead the regulated gait pattern by using the characteristics of grovel entrainments between CPG of lower limbs [13-15] and external stimulation, which let the motion motivated passively. Therefore the trainee is able to learn the regulated motion effortlessly. However, these kinds of locomotion support require place and time for the training, separated from the compensation for locomotive function during daily life using these methods.

This paper introduces the Walk-Mate gait support device [16,17] with these points in mind. Walk-Mate is being developed as a device for simultaneously realizing both locomotion compensation and gait rehabilitation training. The system generates a model gait rhythm in response to the user’s locomotion in real-time, and presents it using auditory stimuli, supporting locomotion by stabilizing the rhythm of foot contacts. In particular, if it is possible for patients themselves to learn comparatively well-regulated gait rhythm using Walk-Mate, it can also be expected that the system will fulfil the requirements for gait rehabilitation training in daily life.

We previously conducted an investigation into the effectiveness of the system as a gait compensation device, and reported that by using Walk-Mate for gait support, asymmetries in step timing and fluctuations during locomotion can be reduced [18]. However, it has not yet been made clear whether the improvements in gait function achieved using Walk-Mate are learned by patients, and whether gait stabilization is retained after support is stopped. In this paper, we therefore compare the results of gait rehabilitation training using Walk-Mate to a previous gait training method using rhythmic auditory stimulation at fixed intervals as a reference. In particular, the objective was to make clear not only the effectiveness of Walk-Mate as a gait compensation technology, but also its effectiveness as a gait rehabilitation training device, and to analyze the process of improvement in locomotion due to gait training using Walk-Mate.

## Methods

**Figure 1:**
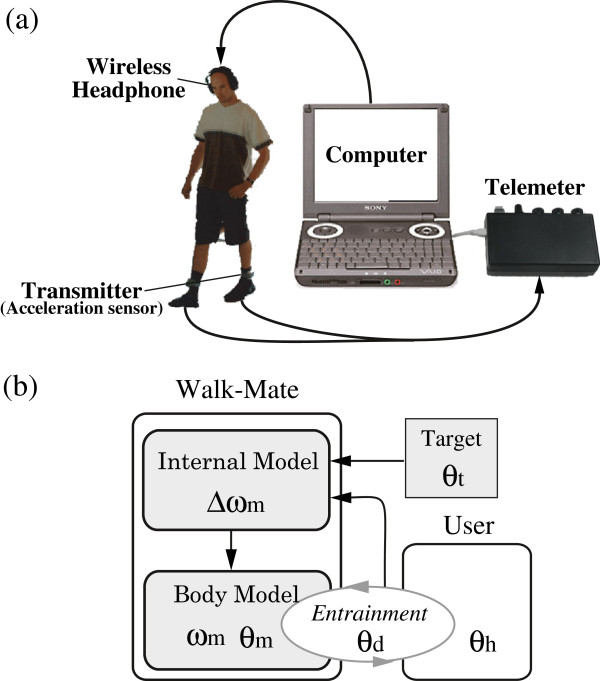
**Interactive gait training device “Walk-Mate”.** Walk-Mate gives rhythmic sound cue according to the user's footstep tempo measured by the acceleration sensor in the Transmitter **(a).** Body Model realize stable phase difference qd by the mutual entrainment with user's footstep rhythm to realize the function of stability of gait rhythm. Internal Model controls characteristic frequency wm by Target phase difference qt and qd to adapt the tempo of the cue to the tempo of the footstep rhythm **(b).**

The Walk-Mate gait support device, which was developed by Smart Sensors Technology Corporation (SSTCORP., Nagano Japan) as an non-commercial-available device under the leadership of Yoshihiro Miyake as a author of this study, uses rhythmic auditory stimulation to improve locomotion rhythm. Users wear a transmitter (WM-1019-023, SSTCORP., Nagano Japan, size: 65 × 75 × 35 mm^3^; weight 143 g × 2; frequency response: 100 Hz) containing a 3-axis acceleration sensor on each ankle in order to detect the instant that each foot contacts the ground, and wireless headphones using the 800 Mhz frequency band while they walk (Figure 1a). The acceleration data sent by the transmitter is forwarded to a compact telemeter (WM-1019-M1C, SSTCORP, Nagano Japan, frequency response: 100 Hz) using the 2.4 GHz band and recorded on a PC (VAIO PCG-U101, Sony, Tokyo, Japan) attached to this device. The absolute value of the vector at each time instant is obtained from the time sequence of recorded acceleration data using the PC. When this value is greater than seven times the value distributed over the preceding 10 seconds, a foot-ground contact is regarded as having occurred. The rhythm control presented to users is based on this information. This kind of locomotion control method providing a rhythm aurally is known as the Rhythmic Auditory Stimulation (RAS) method. It has been reported that rhythmic auditory stimulation at between 1- and 2-second fixed intervals is effective in improving the locomotion of patients with Parkinson’s disease and stroke patients [9,10]. In this aspect, Walk-Mate expands on these methods by using real-time rhythm control for subjects in response to their gait condition, and is capable of providing the functions of a gait compensation device. 

In addition, this rhythm control is conducted according to the so-called “Dual-Dynamics model” [16,17] (Figure 1b). This Dual-Dynamics model is consisted of Body model and Internal model, which processes control the timing of presenting rhythmic sound. The body model is constructed using the non-linear oscillator. By assuming a phase oscillator for the CPG (Central Pattern Generator) controlling locomotion, the objective is taken as achieving the stable entrainment of the rhythm between the user’s footstep and the oscillator. In order to cause the phase difference of the user’s ground-contact rhythm defined in the body model to converge to the target value θt, the natural frequency of the of the body model ωm is controlled with an Internal Model. For the detail of “Dual-Dynamics model”, please refer [17].

Furthermore, for the actual Walk-Mate system, these models are implemented with left-right independence, and the sounds presented may also use different tones on the left and right. This is because by setting the target phase differences in the internal model, θt, to different values on the left and right, the timing of the sounds presented based on the instants of the user’s ground-contacts may take different values on the left and right. For example, when the target phase difference for the unimpaired side is 0 rad, and the target phase difference for the paretic side is −0.3 rad, Walk-Mate’s internal model presents a rhythmic sound in synchrony with the user’s ground contacts on the unimpaired side. On the paretic side the rhythm of sounds presented to the user is 0.3 rad ahead of the ground-contact instants, constituting stimulated low-level timing control. It has been reported in previous research that for hemiparetic patients with gait impairment there is a tendency for the swing phase of the paretic side to be significantly longer than that of the unimpaired side [11,19,20]. This indicates that the instant the swing phase of the paretic side completes (*i.e.,* the timing of the ground contact of the paretic leg) has a tendency to lag in phase relative to the unimpaired side. By setting the hemiparetic target phase difference to a negative value, it becomes more phase-leading, and results alleviating the left-right asymmetry in the leg-motion pattern may be expected.

Meanwhile, by achieving entrainment between the user’s gait rhythm and the body model, and adapting the stimulation rhythm to the user’s gait tempo, the capability of stabilizing the user’s gait tempo itself may be simultaneously realized. Under this form of stimulation, users may achieve their appropriate stable gait tempo by simply matching the timing of the ground contacts during their own leg motion pattern with the emitted rhythmic sounds.

By operating these two types of control mechanism simultaneously, Walk-Mate reduces left-right asymmetries and fluctuations in the gait tempo, providing gait support with the objective of improving the regularity of locomotion.

The objective of the experiments conducted was to investigate Walk-Mate, which has thus far been used mainly as a gait compensation device, and instead examine its effectiveness as a gait-training device. Walk-Mate fulfils the functions of a gait-compensation device, and the mechanism for presenting rhythm stimuli in response to patients’ gait rhythms constitutes a special characteristic. The most important focus of the experiments was considered to be revealing the effectiveness of Walk-Mate as a gait-training device by clarifying the relationship between the presence or absence of such a mechanism and the results of gait training. Training using Walk-Mate was investigated with this point in mind, but more significantly, Walk-Mate was validated by comparison with a control group that used a previous gait training method which presents constant rhythmic stimulation but does not include mutual interactions. Also, in order to evaluate the effectiveness of both for gait compensation and gait training, a continuous 5-day training period was established, and the differences between locomotion before, during and after training were investigated.

Stroke patients with lower extremity motor impairment were selected as subjects, divided into two groups (Walk-Mate group, and control group) and training was conducted accordingly (see Table 1). All subjects had cranial nerve damage associated with strokes, and motion impairment associated with hemiparesis, as reported by their attendant physicians and according to clinical examinations by doctors participating in the experiment. The Walk-Mate group comprised 8 subjects (5 males, 3 females) with an average age of 57.88 ± 11.93 years, a Berg balance [21] score of 48.50 ± 5.68, and a Barthal index [22] score of 68.50 ± 27.0. The control group also compromised 8 subjects (5 males, 3 females) with an average age of 56.75 ± 16.14 years, a Berg balance score of 42.63 ± 7.65, and a Barthal index score of 68.13 ± 27.12. Regarding the ages, and two impairment indices, no significant difference between the two groups was found (age: *p* = .47, duration of stroke: *p* = .35, Berg balance score: *p* = .28, Barthal index score: *p* = .39, Student t-test). In addition, all subjects, although unstable, were capable of walking independently with the aid of walk-assisting devices such as walking frames or walking sticks. None of the patients had prior experience of gait training using auditory rhythm stimulation or Walk-Mate. 

**Table 1:** Subjects’ profiles

**Subject**		**Sex**	**Age**	**Paretic side**	**Dulation of Stroke (month)**	**Barg Barance**	**Bacthal index**
**Walk-Mate condition**	**A**	**Male**	**52**	**Right**	**18**	**49**	**85**
	**B**	**Female**	**43**	**Left**	**9**	**48**	**95**
	**C**	**Male**	**42**	**Right**	**11**	**43**	**95**
	**D**	**Female**	**55**	**Right**	**10**	**32**	**70**
	**E**	**Male**	**59**	**Right**	**2**	**46**	**40**
	**F**	**Male**	**59**	**Left**	**0**	**49**	**40**
	**G**	**Female**	**73**	**Right**	**1**	**52**	**30**
	**H**	**Male**	**77**	**Left**	**44**	**54**	**75**
**Control condition**	**I**	**Male**	**52**	**Left**	**56**	**39**	**85**
	**J**	**Male**	**49**	**Left**	**21**	**56**	**95**
	**K**	**Male**	**40**	**Right**	**20**	**51**	**95**
	**L**	**Female**	**45**	**Left**	**18**	**35**	**95**
	**M**	**Male**	**66**	**Left**	**1**	**49**	**25**
	**N**	**Female**	**88**	**Left**	**2**	**35**	**45**
	**O**	**Female**	**50**	**Right**	**2**	**49**	**75**
	**P**	**Male**	**67**	**Left**	**1**	**42**	**45**

In each of the experiments subjects were equipped with a Walk-Mate transmitter, and walked at a steady speed in a normal manner around a 20 m circular track in a quiet room with the impaired side away from the center. If patients experienced fatigue during the experiment, reported physical unease or a desire to take a break, some minutes of rest were permitted. Behavior such as talking during the experiment was prohibited in advance, insofar as patients did not require to report physical unease, and in fact this did not occur during any of the experiments. Moreover, each of the subjects indicated that they themselves felt very comfortable with this form of walking. All of the sessions were conducted twice a day, and continuously for 5 days. For the first session on the first day of the experiment however, each patient performed only 300 seconds of walking independently in order to measure their initial gait condition. An identical 300-second evaluation of their walking was conducted on the day following the last day of experimentation, in order to evaluate the effectiveness of the training.

The subjects were therefore involved in a total of 9 training sessions conducted from the second session on the first day, to the second session on the fifth day. However, some subjects who complained of physical unease rested during 1 or 2 training sessions (see Table 1). During training, in order for subjects to clearly and independently discriminate the rhythm presented for the left and right sides, they wore wireless headphones during walking. At this time, the first 10 seconds of locomotion were considered as a process during which subjects selected their pace of locomotion and no sound was presented. In addition, after confirming in advance that subjects could clearly perceive the rhythm presented using sounds for the left and right sides, they were requested to focus on the rhythmic sounds, and coordinate their leg motion with it while walking.

For the Walk-Mate subject group, the left and right target phase difference was set to 0 rad on the unimpaired side, and the impaired side was set for each patient to the average value of the left-right phase difference in foot contact timing over the whole range of motion measured during the first session on the first day. When this target phase difference was larger than the actual asymmetry, the feedback gains in the internal model are too large due to the possibility that the auditory tempo stimulation based on the body model may not achieve stability.

Regarding the subjects in the control group using constant tempo stimulation, the average tempo for each subject obtained from the data measured on the first session of the first day was increased by 5% and presented as a rhythm stimulus. This setting was based on the RAS method used by Thaut [11], according to which a tempo 5-10% faster than the average rhythm is presented.

The measurement of locomotion was accomplished entirely using the transmitters containing acceleration sensors attached to the subjects’ ankles. The subjects’ foot-ground contact instants were identified based on the data transmitted in real-time every 10 ms.

As previously stated, Walk-Mate does not merely function as a gait compensation device, but is also expected to be capable of improving gait as a gait rehabilitation training device. In this analysis an evaluation of the gait support provided by Walk-Mate was conducted during locomotion and also after 5 days of such support, using gait training methods that present fixed-rhythm stimuli as a reference in order to validate Walk-Mate’s effectiveness. In particular, the two targets of “alleviating asymmetries in foot contact timing” and “reducing contact-tempo fluctuations” expected from Walk-Mate gait support are established and evaluated.

Left-right asymmetries in the rhythm of leg motion can be defined in terms of the left-right phase difference due to the contact timing the feet. In particular, when the contact timing of the feet is absolutely symmetrical, the phase difference of the left and right feet is in anti-phase, *i.e.,* contacts occur with an offset of ± πrad. However, when the contact time of one foot lags in phase behind the other, the phase difference becomes larger. From this perspective, the asymmetry occurring between the presentation of the first stimulus at time t_1_ and the final stimulus at time t_n_ can be defined as shown in Equation (3) below, as an average of the measured absolute values of the deviation from an anti-phase condition between the feet each time a foot contacts the ground.

(1) $$Asymmetry_{t_{1}}^{t_{n}}=\frac{2\pi }{2\left(n-1\right)}\cdot \sum_{k=1}^{n}\left|\frac{t_{p\_k}-t_{h\_k}}{t_{p\_k}-t_{p\_k-1}}-\frac{t_{h\_k}-t_{p\_k+1}}{t_{h\_k}-t_{h\_k-1}}-1\right|$$

t_p_n_, and t_h_n_ are the contact times of the impaired and unimpaired sides. It is assumed that t_p_n_ > t_h_n_ > t_p_n+1_.

As stated above, it is known that automatic control based on self-exciting rhythm generating mechanisms existing in the spinal column known as central pattern generators (CPGs) are deeply involved in the control of human motion tempo, which is also known to have a uniform stability. However, damage to the nervous system, *etc.*, can reduce the dynamic stability of these rhythms [23]. It has also been suggested that this kind of stability reduction may be a trigger for the causes of serious accidents such as falling. The fluctuation in gait tempo is defined accordingly in terms of the standard deviation of the ground-contact period during leg motion, and this index is used to evaluate fluctuation.

(2) $$Fluctuation_{t_{1}}^{t_{n}}=\sqrt{\frac{1}{n-1}\cdot \sum_{k=1}^{n}{\left(Period_{p\_k}-ave\_Period_{p}\right)}^{2}}$$

Here, $Period_{p\_k}=t_{p\_k}-t_{p\_k+1}$, and $ave\_Period_{p}=\frac{1}{n}\cdot \sum_{k=0}^{n}\left(t_{p\_k}-t_{p\_k+1}\right)$.

## Results

In this section the effects on locomotion owing to Walk-Mate and the fixed-rhythm stimulation gait support method are analyzed with regard to left-right asymmetries in foot-ground contact timing during walking and fluctuations in cycle time.

### Changes in ground-contact timing asymmetries

**Figure 2:**
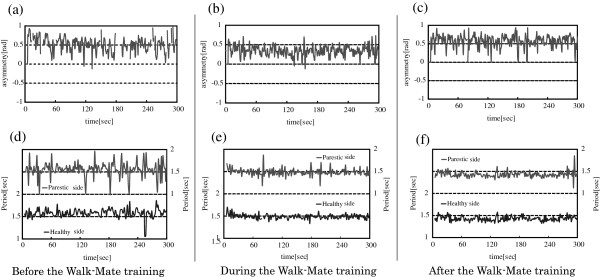
An example of gait training process with Walk-Mate.

Changes in asymmetries of foot-ground contact timing owing to Walk-Mate and fixed-rhythm stimulation gait-training prior to, during and following training were analyzed. Figure 2 (a), (b), and (c) shows the data for subject H prior to gait training using Walk-Mate (session one on day 1), during training (session two on day 3), and after all the sessions had been completed (the day after the final session).

Comparing (a) and (b) in Figure 2, the values for asymmetry are different before and during training and it can be seen that asymmetry appears close to zero when Walk-Mate is active, indicating the benefits that bring this subject’s locomotion towards a symmetric state. However, it is not possible to confirm that asymmetry was reduced after training (c), by comparison to the data prior to training (a). This means that while using Walk-Mate, asymmetry was improved, but this result was not retained after training was completed.

**Figure 3:**
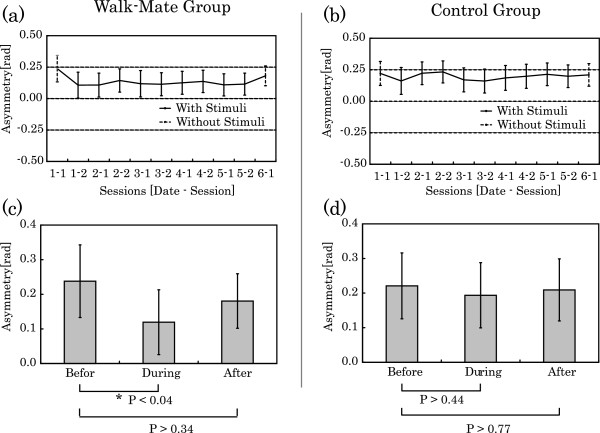
Training processes with Walk-Mate training and with constant auditory stimuli.

To survey this trend in both the Walk-Mate and control groups, the group average during each session was computed, and shown as a time series in Figure 3. As a result, from (a) in Figure 3, the Walk-Mate group demonstrate a significantly smaller value during training than before training (F(9,63) = 2.13, P < 0.04, one-way repeated ANOVA). Asymmetry during training with Walk-Mate was significantly reduced by comparison to the initial training session (Figure 3(a)1-2), and it can be seen that this trend was maintained over subsequent sessions. However, asymmetry after training is greater than during training, and a significant difference compared to before training was not observed (F(9,63) = 1.16, P > 0.33, one-way repeated ANOVA). In fact the value prior to beginning training is closer. For the control group on the other hand, the lowest value for asymmetry may be seen in Figure 3(b)1-2, but it is not significant with respect to the initial training session (F(9,63) = 1.37, P > 0.22, one-way repeated ANOVA). During training in subsequent sessions, and after training no significant change was observed (F(9,63) = 1.37, P > 0.22, one-way repeated ANOVA).

In order to confirm these trends more accurately, the average values of these data are shown in Figure 3(c). This confirms that there is a significant difference for the Walk-Mate group before and during training (P < 0.04, Student t-test). Thus, Walk-Mate interaction improved asymmetry, but also that improvements were not retained after training, based on the finding that a significant difference between asymmetry prior to training and following training could not be confirmed with Walk-Mate (P > 0.34, Student t-test). For the control group on the other hand, comparing the values before and during training does not reveal a significant change (P > 0.44, Student t-test), and neither was there a significant change in the asymmetry values following training (P > 0.77, Student t-test). This means that for the gait training method with fixed-rhythm auditory stimulation used in this experiment, improvement in asymmetry may have been achieved but only on a temporary basis and were not retained after training.

As neither group showed a significant change in asymmetry values before and after training, it seems that the training methods examined are not adequate rehabilitation methods for improving asymmetry. However, comparison of the data for both groups prior to and also during training suggests that while presenting stimuli with a constant rhythm does not provide satisfactory compensation for asymmetric locomotion, gait training using Walk-Mate does.

### Changes in gait tempo fluctuations

This section analyzes gait training based on Walk-Mate and fixed-rhythm stimulation in a similar manner to the previous section, but regarding changes in fluctuations of foot-ground contact tempo. An example set of data may be seen in Figure 2 (d), (e) and (f) for subject H prior to gait support (session one on day 1), during training (the session two on day 3), and after all sessions had been completed. Comparing Figure 2 (d) and (3), the fluctuation of the gait ground-contact cycle which corresponds to the amplitude of fluctuation in the data shown in the graph is different before training and during training, and a trend of reduction can be seen when using Walk-Mate. In addition, a trend of reduction can also be seen when comparing the amplitude of fluctuations after training with those before training. This means that when using Walk-Mate, not only are the fluctuations in locomotion improved, but these results are also retained after training is complete.

**Figure 4:**
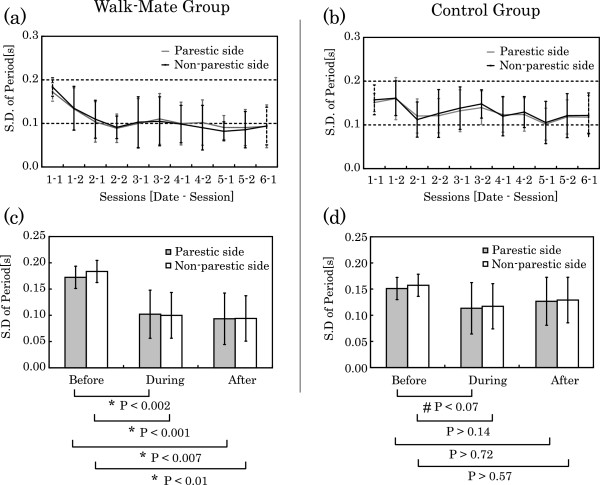
Temporal developments of footstep period with the training process of Walk-Mate training and with constant auditory stimuli text.

Since this trend may be observed in both the Walk-Mate group and the control group, the average data for both groups over each session was computed, and the resulting time series is shown in Figure 4. In Figure 4 (a), it can be seen that for the Walk-Mate group the value is significantly smaller during training than before training (unimpaired side: Pχ2 (9, N = 63) = 24.33 P < 0.004, impaired side: Pχ2 (9, N = 63) = 24.33 P < 0.002, Friedman test). It can be seen that there is a trend for reduction during gait training with Walk-Mate. In addition, no significant change can be seen between the values after training is completed, and during training (unimpaired side: Pχ2 (9, N = 63) = 10.20 P > 0.20, impaired side: Pχ2 (9, N = 63) = 12.16 P > 0.33, Friedman test). It was thus observed that the reduced trend was retained after training.

For the control group, comparing the value before training and during training reveals a significant reduction (unimpaired side: Pχ2 (9, N = 63) = 18.60 P < 0.03, impaired side: Pχ2 (9, N = 63) = 19.53 P < 0.02, Friedman test). The trend for reduction can therefore also be observed during training that presents fixed-rhythm stimulation. However, regarding the completion of training, the significant reduction could only be observed during training (unimpaired side: Pχ2 (9, N = 63) = 14.67 P > 0.10, impaired side: Pχ2 (9, N = 63) = 16.77 P > 0.07, Friedman test). It was thus observed that the trend was not sufficiently retained after training.

In order to confirm these trends more accurately, the average values of these data are shown in Figure 4 (c). This confirms that there is a significant difference for the Walk-Mate group before and during training (unimpaired side: z = 4.09, P < 0.001, impaired side: z = 4.16, P < 0.001, Mann–Whitney U-test). Thus, the Walk-Mate interaction alleviated fluctuations during locomotion. In addition, this significant difference was also confirmed between the state prior to and following training (unimpaired side: z = 2.56, P < 0.01, impaired side: z = 2.58, P < 0.007, Mann–Whitney U-test). It may therefore be concluded that improvement of fluctuations during locomotion achieved through interaction with Walk-Mate were retained.

For the control group, comparing the leg motion on only the impaired side prior to training and during training reveals a significant difference (unimpaired side: z = 1.68, P > 0.14, impaired side: z = 1.79, P > 0.07, Mann–Whitney U-test). A significant change in the value reflecting the fluctuation of gait period throughout training was not sufficiently demonstrated. However, in the previously stated evaluation of the time series variation, a significant trend for reduction in fluctuations during training was observed. This indicates that the trend towards reduction in fluctuations during training is not sufficient over all, but may be thought of as a gradual strengthening that occurs while training advances. In addition, comparing the values before and after training, no significant difference was observed (unimpaired side: z = 0.56, P > 0.57, z = 0.36, impaired side: P > 0.72, Mann–Whitney U-test). This indicates that for the fixed-rhythm sound-stimulation gait training method used, somewhat beneficial results that improved the fluctuations during locomotion were observed during training, but these results were not retained after training.

Only the Walk-Mate group showed a change in values reflecting the amplitude of gait fluctuations before and after training, suggesting that training using Walk-Mate not only functions for gait compensation, but also effectively for rehabilitation training to improve fluctuations in locomotion. At the same time, the results for the control group revealed only a modest reduction in fluctuations of the gait period during training, suggesting that the training provided a modest compensatory result regarding fluctuations during training and no beneficial results after training.

## Discussion

It was revealed that improvement of asymmetry in ground-contact timing during locomotion could be obtained through interaction with Walk-Mate. In addition, it was made clear that these results could not be obtained with previously proposed training methods using fixed-rhythm aural stimulation. However, these results could not be confirmed by evaluations made after training was completed using Walk-Mate or the previously proposed method. This revealed that gait compensation improving such gait asymmetries could only be achieved using Walk-Mate for gait support. The results therefore suggest that interactive gait training methods facilitate comparatively advanced gait compensation functionality, but that gait training using aural stimulation for 5 days was not adequate as rehabilitation training for improving the asymmetries in locomotion.

Regarding the fluctuations in foot-ground contact timing, interaction with Walk-Mate did not merely achieve temporary benefits, as the results were retained after the completion of training. In comparison, training using fixed-rhythm stimulation achieved only a modest benefit regarding fluctuations, and only during training. This suggests that the interactive gait training used in Walk-Mate was responsible for the gait compensation results of stabilizing the foot-ground contact period, and the results of rehabilitation. In addition it was not possible to obtain satisfactory results for improving gait-tempo fluctuations using the gait-training method with fixed-rhythm stimulation. This suggests that this kind of training using a fixed rhythm is not capable of achieving sufficient gait compensation, nor gait rehabilitation. The interactive mechanism of Walk-Mate was shown to be effective for both gait compensation and gait rehabilitation aimed at improving gait asymmetry and fluctuations.

For gait training that presents a rhythm stimulus, the stimuli fulfil the role of a pace maker setting the rhythm of the gait. It has been shown that self-excitation rhythm generating mechanisms in the spinal cord, or CPGs, show strong involvement in the control of gait rhythm [24]. Therefore, in order to regulate the gait rhythm generated by the CPG, it is necessary to establish a coherent relationship with the presented rhythm, and the relationship with the stimulus must be settled in a stable manner. However, to construct such a coherent relationship, it is also necessary that a large difference does not exist between the dynamic characteristics of the presented stimuli and the inherent dynamic characteristics of the patient’s gait rhythm. However, since the dynamic characteristics of the rhythms generated by the CPGs of the present subjects were constrained by muscle stress, *etc.*, caused by hemiparesis, it was predicted that they would have problems controlling the dynamic characteristics of locomotion themselves in response to the stimuli presented. In particular, when fixed-rhythm stimuli were presented, it was necessary to make it possible for patients to unilaterally adapt the dynamic system of their own legs to the stimuli and generate a fixed gait rhythm. At the same time, when the rhythm was controlled in response to a patient’s gait in the manner of Walk-Mate, it was not necessary to control these types of dynamic characteristic, so it was thought that for handicapped persons, a coherent relationship could be automatically constructed in a comparatively easy fashion. In practice, since a fixed-rhythm stimulus does not contain fluctuations, in order to generate a coherent relationship with the stimulus, it is necessary for the gait rhythm itself to achieve a similar state with few fluctuations. However, by comparison with Walk-Mate, the standard deviation of the gait cycle during training is 22% larger, and it was thought that patients were unable to establish a coherent relationship as a result. A related difference in the improvements to locomotion can therefore be predicted, based on the difference in difficulty of adapting to the stimuli. These factors suggest that interactive gait training using Walk-Mate is superior at improving gait rhythm by comparison to fixed-stimulus methods. They also support the result that Walk-Mate is an effective gait compensation system.

The results also indicated a difference in the improvements regarding both the left-right asymmetries in ground-contact timing, and the fluctuations in the ground-contact period. Regarding the asymmetries, a trend of improvement was only observed during interaction with Walk-Mate. It is thought that these asymmetries are caused by differences in the inherent dynamic characteristics of gait rhythm caused by differences in muscle tone of the left and right legs due to hemiparesis. However, when cyclic dynamic systems like the motion of the legs experience interference from an external rhythm with approximately the same natural period like Walk-Mate, it is possible to achieve entrainment of gait rhythm [25,26]. For this reason, it is possible to regulate the phase characteristics of the rhythm without making unreasonable changes to the natural dynamic system of leg motion rhythm. In particular, during interaction with Walk-Mate, since the natural frequency of the rhythm presented to patients is adjusted in accordance with the target phase difference, it is possible to present a rhythm with a somewhat faster timing than that of ground contact. In this way, people may advance the phase of the rhythm making it symmetric without having to make large changes to their dynamic characteristics. However, since it is assumed that for these kinds of adjustment the stimuli are always presented continuously, the improvements regarding asymmetry cannot be expected for unassisted walking insofar as the natural dynamic system itself does not change. It is therefore be proposed that the reason the resulting improvements were not observed after training was that the training was achieved based on the lower order nervous systems central to CPGs, and was insufficient to achieve a change in the natural dynamic system for which an improvement in muscle tone necessary.

Regarding the fluctuations in ground-contact period however, an improvement was not only observed during interaction with Walk-Mate, but also afterwards. It is therefore thought that in addition to changing the rhythm through entrainment, training also yielded changes in the fluctuation characteristics of the dynamic system caused by the gait rhythm. In particular, it is thought that this kind of ground-contact-period fluctuation is caused by instabilities in the dynamic system of leg motion brought about by left-right discrepancies in muscle tone. It is therefore thought that relatively high-level motion control mechanisms at the cerebellum and cortical level involved in the control of the dynamic characteristics of leg motion [27,28] have a significant influence on the emergence of this kind of fluctuation. In particular, it has been shown that the cerebellum is deeply involved in the learning of limb motion patterns [29], and it is proposed that learning using relatively high-level motor nerve systems including the cerebellum brought about the improvements in locomotion both during and after training. For the control group however, there was no significant improvement during or after training in comparison to the state before training, but a significant trend for progress regarding the fluctuation phenomenon was observed during training. This suggests that these changes were mainly achieved through the coherence of rhythm entrainment based on motion control at the CPG level, and the training using fixed-rhythm stimuli was insufficient to change the dynamic characteristics.

These factors suggest that the differences observed in the improvements regarding left-right asymmetry and fluctuations were achieved for these gait disorder characteristics through motion control systems at different levels. They also suggest that the dynamic characteristics related to improvement of fluctuations, by comparison to those of the asymmetries, tend to improve more quickly.

## Conclusions

This research conducted gait assistance experiments using the Walk-Mate device, which is able to present a target gait tempo based on a user’s foot-ground contact rhythm using sound. As a result, it was made clear that interaction with Walk-Mate is effective in improving asymmetries of gait rhythm, improving fluctuations in the ground-contact period, and also that these benefits cannot be satisfactorily achieved by presenting fixed-rhythm stimuli, as the former studies methods[10,11]. In addition, there is a tendency for the resulting improvement of gait fluctuations to be retained after gait training, suggesting that 5 days of training with Walk-Mate was also effective as rehabilitation training.

Therefore this result suggest not only that Walk-Mate we have proposed as gait compensation device can realize function to support gait training, but also that the training by Walk-Mate may realize higher effect of the training in a part. In concluding, we may say that, being compatible with gait compensation, the training with Walk-Mate we have suggested is more beneficial for the hemiparetic stroke rehabilitation in a part of the training effects.

In our research to date, phase control of the gait rhythm for a coordinated gait has been demonstrated using a hierarchical control mechanism of locomotion, and through observation of both gait coordination mediated by footstep sounds from two people and the process of gait coordination with Walk-Mate [17,30]. It has also been shown that this kind of hierarchical control mechanism may be involved in the temporary improvement of locomotion [31]. The results of this research suggest that mutually interactive gait rhythms based on this kind of hierarchical control are effective for achieving results in locomotion rehabilitation, and reveal that hierarchical control based on dynamic mutual interaction with the environment may be involved in the emergence of new motor function. Furthermore, the relationship between mutual interaction with the environment and the emergence of new functionality has been discussed in the fields of cognitive therapeutic exercise [32,33], music therapy [34,35], and infant development [36], but this is the first research to specifically demonstrate the effectiveness of mutual interaction during gait rehabilitation.

From now, more detailed gait analyses are planned in order to evaluate the effectiveness of the Walk-Mate rehabilitation technology. Concretely, gait training is planned for a period of 6 weeks, as used in previous research, and the results will be compared to training using a fixed rhythm. The possibility that asymmetries and fluctuations in gait rhythm are brought about at different levels of motion control has now been made clear, and it is therefore planned to further elucidate the improvement mechanisms for each according to new experiments.

Also the detail relationship between the training effects of Walk-Mate and the clinical evaluation was not dealt because the primary purpose of this study is to clarify whether the Walk-Mate training is effective for real hemiplegic gait. Therefore, from now on, more suitable selection of target patients will be needed to discuss the training effect from the neurological point of view. For examples of typical indexes for the selection, we can suppose the time to the duration of Stroke and diary on the number of falls.

It has been shown according to the evaluation of gait training based on rhythm stimulation that in addition to the rhythm characteristics like those used in this research, the analysis of leg motion patterns, and observation of spatial characteristics such as changes in gait speed or stride length are also of value [11]. It is intended to introduce these indicators and perform analyses from a spatial perspective. It has been suggested that training with this kind of rhythm presentation is useful not only for stroke patients, but also cases of Parkinson’s disease and Huntington’s disease [37]. The application of Walk-Mate to such alternative cases of gait training and the investigation of its effectiveness is also under consideration.

## Competing interests

The authors declare that they have no competing interests.

## Authors’ contributions

TM contributed to the design of the study, conducting of the experiments, the software development and the interpretation of the results. BH and JH contributed to the design of the study, to the assessment of patients, to the acquisition of data and to its interpretation. YM and EP contributed to the design of the study, to the financial support of this study. All the authors have revised the manuscript and have given their final approval for publication.

## Author details

Author details^1^GRP - Generation Research Program, Humanwissenschaftliches Zentrum, Ludwig-Maximilians-Universitat München, Prof.-Max-Lange-Platz 11, 83646,Bad Tölz, Germany. ^2^Tokyo Institute of Technology, G3-821, 4259, Nagatsuta, Midori-ku, Yokohama, Kanagawa 226-8502, Japan. ^3^Aoyama Gakuin University, 5-10-1 O-505b, Fuchinobe, Chuo-ku, Sagamihara, Kanagawa 252-5258, Japan. ^4^EKN - Entwicklungsgruppe Klinische Neuropsychologie, Abteilung fuer Neuropsychologie, Krankenhaus München-Bogenhausen, Dachauer Strasse 164, D-80992, Munich, Germany. ^5^Technische Universität München, Connolly strasse. 32, 80809, Munich, Germany. ^6^Institute for Medical Psychology, Ludwig -Maximilians-Universitat München, Goethestrasse 31, D-80336, Munich, Germany.
